# The Post-Transcriptional Regulatory Element of Hepatitis B Virus: From Discovery to Therapy

**DOI:** 10.3390/v16040528

**Published:** 2024-03-29

**Authors:** Karim Mouzannar, Anne Schauer, T. Jake Liang

**Affiliations:** Liver Diseases Branch, National Institute of Diabetics and Digestive and Kidney Diseases, National Institutes of Health, Bethesda, MD 20892, USA; anne.schauer@nih.gov

**Keywords:** hepatitis B, RNA, PRE, ZCCHC14, TENT4A/B, RNA tailing, therapeutics

## Abstract

The post-transcriptional regulatory element (PRE) is present in all HBV mRNAs and plays a major role in their stability, nuclear export, and enhancement of viral gene expression. Understanding PRE’s structure, function, and mode of action is essential to leverage its potential as a therapeutic target. A wide range of PRE-based reagents and tools have been developed and assessed in preclinical and clinical settings for therapeutic and biotechnology applications. This manuscript aims to provide a systematic review of the characteristics and mechanism of action of PRE, as well as elucidating its current applications in basic and clinical research. Finally, we discuss the promising opportunities that PRE may provide to antiviral development, viral biology, and potentially beyond.

## 1. Introduction

Chronic hepatitis B (CHB) is a major global health problem that affects an estimated 296 million people with a high risk of liver-related morbidity and mortality, notably cirrhosis and hepatocellular carcinoma (HCC) [1]. Current therapies rarely cure CHB due to their failure to eradicate HBV covalently closed circular DNA (cccDNA). This persistent form of the HBV genome resides in hepatocytes and constitutes the viral reservoir liable for viral relapses [2]. In addition to the currently approved interferons and nucleoside analog therapies, several new HBV therapeutic strategies are currently in development, spanning from late preclinical to advanced clinical phases, with the aim to achieve a better functional cure [3,4,5].

**Figure 1:**
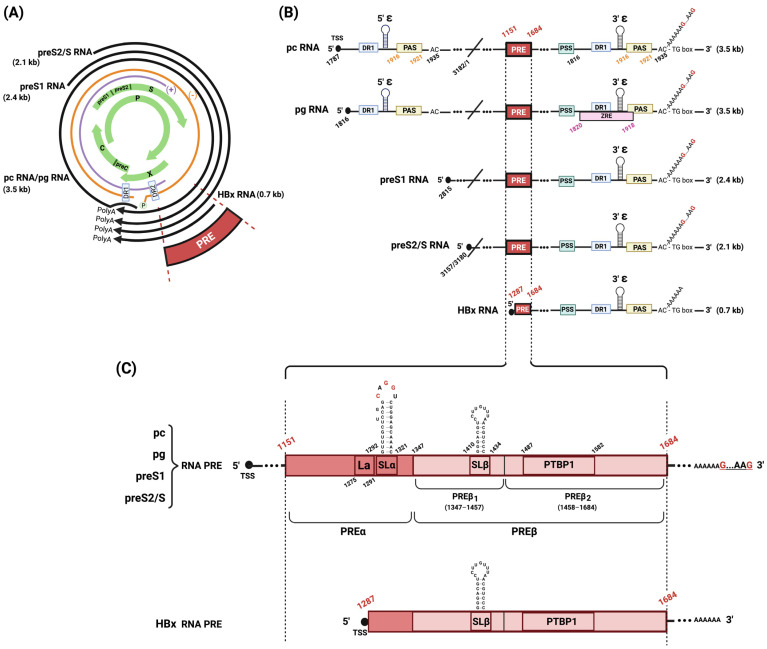
Schematic representation of HBV genome organization, transcripts, and PRE bipartite structure. (**A**) HBV genetic map. HBV rcDNA is depicted with its negative (orange) and discontinuous positive (purple) strands, DR1/DR2 intersection, and covalently attached HBV polymerase (P). The four overlapping open-reading frames (preC/C, P, PreS1/preS2/S, and X) are shown (green arrows). Four classes of transcripts generated by HBV that terminate at a shared polyadenylation site (polyA) are shown (black arrows). (**B**) HBV transcripts. The five HBV RNA transcripts are shown, each depicting the transcription start site (TSS) at the 5′ cap termini (black circles), and several viral cis-elements: PRE, polyadenylation stimulating sequence (PSS), unconventional UAUAAA poly(A) signal (PAS), and ZAP-responsive element (ZRE). Nucleotide numbering is based on HBV genotype D. (**C**) PRE bipartite structure. An expanded view of PRE shows that all HBV RNA transcripts contain a complete PRE, except for HBx mRNA. Complete PRE is composed of PREα and PREß (containing elements PREß1 and PREß2). PREα has a La protein binding site (La) and a conserved stem–loop structure (SLα). PREß1 has a stem–loop structure (SLß). PREß2 has a polypyrimidine binding protein 1 docking site (PTBP1). Figure was created with Biorender.com.

The life cycle of HBV is characterized by several key steps. The initial step is the entry into hepatocytes marked by the attachment of the HBV envelope protein to the human Na^+^-taurocholate co-transporting polypeptide (NTCP) [6]. Following entry, the capsid undergoes uncoating and trafficking to the nucleus, where HBV relaxed-circular DNA (rcDNA), which is a partially double-stranded DNA, is converted into cccDNA. This episomal DNA is then structurally organized into a mini-chromosome by histone and non-histone proteins and functions as the template of viral transcript synthesis [7,8]. The transcription of cccDNA from different promoters results in the production of five HBV RNAs: the 3.5 kb pre-core RNA (pc mRNA) and pre-genomic RNA (pg RNA), the 2.4 kb large surface protein RNA (preS1 mRNA), the 2.1 kb middle/small surface protein RNA (preS2/S mRNA), and a 0.7 kb HBx mRNA (Figure 1A).

Although pc and pg RNAs are two genome-size HBV transcripts, they have different outcomes. The pg RNA has two functions. First, it serves as bicistronic mRNA for the core protein (C) and HBV polymerase (P). Second, it is encapsidated with P and reversely transcribed into new rcDNA. On the contrary, at its N-terminal, the pc RNA contains 29 additional nucleotides than the pg transcript. This leads to the translation of HBV E antigen protein (HBe) that has different characteristics than C [9].

After translation of HBV transcripts, the infectious cycle is completed by the HBV surface protein (HB) envelopment of newly formed nucleocapsids containing mature rcDNA. Infectious viruses are then secreted outside hepatocytes through the multivesicular body (MVB)-associated endosomal sorting complex required for transport (ESCRT) machinery.

Here, we review a specific step in the HBV life cycle that is the post-transcriptional regulation of its transcripts. HBV RNAs contain a post-transcriptional regulatory element (PRE) which plays a crucial role in ensuring their stability and nuclear export. HBV PRE (HPRE) facilitates the recruitment of host complex proteins that post-transcriptionally regulate viral RNAs to enhance their expression. In this review we discuss PRE’s structural characteristics, function, mode of action, and potential in HBV therapies and other clinical applications.

## 2. Discovery and Implication of PRE in HBV Life Cycle

There exist critical checkpoints that control the gene expression of cellular mRNAs in mammals, among them, splicing and nuclear export. These mechanisms ensure that only successfully processed mRNAs will be translated. RNAs that do not match the cellular host requirements end up being degraded. A common pathway for host mRNA degradation starts with the removal of the poly(A) tail by deadenylases, de-capping, and finally RNA shortening (3′ to 5′ or the opposite) by exoribonucleases [10,11,12].

In the case of HBV transcripts, abnormal RNA splicing by the host machinery can be deleterious and must be avoided at all costs by the virus to preserve the steady-state level of its transcripts. Also, HBV mRNA is polyadenylated by a non-conventional polyadenylation signal (PAS) element, UAUAAA, which is different from the common AAUAAA present in the majority of all cell mRNA [13]. This PAS may not confer complete polyadenylation required for its stability [14]. Therefore, a necessary post-transcriptional regulatory step occurs to ensure stabilizing HBV transcripts and their export to the cytoplasm for translation. This mechanism was discovered to rely on a specific, complex cis-element region, located in the 3′ terminal of all HBV viral transcripts, and called PRE.

PRE was first discovered in 1993 by researchers that studied gene expressions of HBV subtype *ADW* (GenBank: D00329.1) [15]. Authors demonstrated that PRE is essential for the high-level expression of viral gene products, initially contributing to the enhancement of RNA nuclear export of viral mRNAs. Subsequently, it was shown to affect the stability of HBV transcripts [15,16,17]. The discovery of PRE highlights a unique and novel feature of HBV that regulates the biosynthesis and degradation of HBV RNAs [18]. It is first important to understand PRE’s structural features.

## 3. Structural Features of PRE

### 3.1. HBV RNAs Is Composed of Distinct Cis-Elements

HBV transcripts possess multiple cis-elements where various RNA-binding proteins (RBP) can directly bind and cause different outcomes to their steady-state levels (Figure 1B).

All five HBV RNAs are 5′ capped and share a similar 3′ end because they all terminate at the same PAS. On this unique PAS, cellular polyadenylating polymerase alpha (PAPα) is recruited for polyadenylation. When based on HBV genotype D (GenBank: U95551.1) for nucleotide (nt) numbering, PAS is located between 1916 and 1921 nt. There are two PAS elements on 3.5 kb HBV pg RNA and pc RNA. Their transcription initiates upstream of the first PAS and proceeds until encountering the PAS again in a circular genome.

This shared 3′ end also contains a 3′ proximal epsilon (ε) stem–loop structure. The proximal ε stem–loop was shown to be essential for the direct binding of the interferon-inducible ribonuclease (ISG20) that degrades HBV RNAs [19]. The 5′ proximal ε stem–loop structure, on HBV pg RNA and not on pc RNA, serves as specific encapsidation signal RNA [20]. The P protein binds to 5′ stem loop and induces a ribonucleoprotein (RNP) complex with C for encapsidation. DR1 and DR2 are direct repeat sequences implicated in rcDNA formation.

Another cis-element present on pg RNA is the ZAP-responsive element (ZRE) corresponding to the terminal redundant region (nt 1820–1918). This element is essential for binding the zinc finger antiviral protein (ZAP). ZAP was identified as an anti-HBV host factor that selectively targets the pg RNA for degradation [21,22]. ZAP direct binding to ZRE is essential for its antiviral activity [23]. This was demonstrated in vitro for the two ZAP isoforms (hZAP-L and hZAP-S) in hepatocellular carcinoma cell lines. ZAP was also shown to be important for interferon-stimulated gene [24]. Interestingly, ZAP has been previously identified as an antiviral factor for a broad range of RNA viruses, including Moloney murine leukemia virus (MMLV) [21], human immunodeficiency virus (HIV-1) [25], xenotropic murine leukemia virus-related virus (XMRV) [26], Japanese encephalitis virus (JEV) [27], Sindbis virus (SINV), Ross river virus (RRV) [28], Ebola virus (EBOV), and Marburg virus (MARV) [29].

The myeloid differentiation primary response protein 88 (MyD88), an essential adaptor in the signaling cascade of the innate immune response [30], was also shown to downregulate HBV RNAs via a post-transcriptional mechanism [31]. The study showed that MyD88 accelerates HBV pg RNA degradation in the cytoplasm through two main mRNA degradation pathways in mammalian cells (5′-to-3′ and 3′-to-5′ mRNA decay pathways) and by inhibiting the nuclear export of HBV preS1 and preS2/S RNAs via PRE. This study identified nt 1804–2454 as a cis-regulatory sequence essential for MyD88-induced HBV pg RNA degradation.

In addition to the elucidated cis-elements present on HBV transcripts, there exists PRE, which is characterized by a specific structure.

### 3.2. PRE Has a Bipartite Structure

In the first study that discovered PRE, the authors tested the impact of the HBV surface antigen (HBsAg) secretion on several HBV constructs of subtype *ADW* (GenBank D00329.1) [15]. The results showed that the 3′ boundary of HPRE is around the nt 1684 position. They showed that the sequence beyond nt 1684 to the 3′ end of HBV genome had no effect on HBsAg secretion. In contrast, the region from (nt 1236 to 1684) was essential, as its deletion markedly impaired secretion of HBsAg. Further truncation showed a sequence from nt 1372 to 1572, which seemed to be important for HBsAg production.

To precisely define the 5′ boundary of HPRE, a group later generated a series of 5′ deletions and concluded that the functional HPRE is composed of 533 nucleotides and is located at the position (nt 1151–1684) of viral transcripts [17]. Furthermore, the authors tested additional constructs and elucidated the existence of sub-elements within that region. Several HBV fragments were tested. They concluded that PRE is a bipartite RNA structure composed of a 5′ PREα sub-element, encompassed by (nt 1151–1346) and a second one, PREß, between (nt 1347 and 1684) (Figure 1C).

### 3.3. The Motifs of PRE Sub-Elements

PREα contains a La-binding motif (nt 1275–1291) [32] and a stem–loop alpha (SLα) (nt 1292–1321) [33], both of which play essential roles in the export of viral RNAs from the nucleus to the cytoplasm and contribute to their stability [17,32,34].

The La-binding motif sequence allows for the binding of a La protein, which contributes to the stability of HBV pg RNA [32,34,35]. Human La protein is a phosphoprotein weighing 47 kDa, belonging to the family of RBP that contains RNA motifs (RRM) [36]. Degradation of the La protein can be triggered by HBV-specific cytotoxic T-lymphocytes (CTL) via interleukin-2 (IL-2) in HBV-expressing cells. This pathway represents a potential mechanism of viral clearance by targeting HBV RNA for degradation by cellular nucleases [37,38].

HBV SLα is characterized by a pentaloop of five nucleotides, CAGGU (in the case of HBV genotype D)*,* predominantly assuming the conformation of the CNGG(N) family loop featuring a single bulged G residue flanked by A-helical regions [39]. The NMR solution structure of HBV SLα was published previously (PDB 2JYM) [39].

PREß consists of PREß1 (nt 1347–1457) and PREß2 (nt 1458–1684), which, together, confer full functional competence [40]. PREß1 contains an RNA stem–loop SLß (nt 1410–1434) [40]. PREß2 contains a polypyrimidine binding protein 1 (PTBP1) binding site (nt 1487–1582) that plays a direct role in the nuclear export of PRE-containing RNA [41].

The two conserved RNA stem–loops, SLα and SLß, are key functional components within HPRE for HBV nuclear export elements [40,42]. SLα is present in all HBV transcripts except for HBx mRNA. The transcription start site (TSS) of HBx mRNA is located within the 5′ PRE region. Therefore, HBx mRNA does not contain a complete PRE sequence, which could potentially explain why the HBx mRNA half-life is shorter than other HBV mRNAs (Figure 1C).

## 4. PRE Mechanism of Action

### 4.1. The DHQ Molecules and TENT4A/B Complex

Since its discovery in 1993, the precise mechanism by which PRE regulates the nuclear export and stabilization of HBV RNAs has remained unknown. In 2018, an antiviral phenotypic screen of around one million small molecule compounds was conducted to identify potential inhibitors of HBsAg secretion [43]. This screen led to the discovery and characterization of RG7834, a novel small molecule from the dihydroxyquinoline (DHQ) chemical series, as a potent inhibitor of HBV pg, pc, preS1, and preS2/S RNAs. The study showed that RG7834 was well tolerated in both in vitro (differentiated HepaRG and primary human hepatocytes) and in vivo models of HBV infection (HBV-infected uPA/SCID mice model).

Later that year, another research group conducted a phenotypic screen, identifying RG7834 as a selective HBV inhibitor and elucidating its mechanism of action [44]. The structure–activity relationship (SAR) and the structure–property relationship (SPR) of the DHQ series were also studied, and the absolute configuration of RG7834 was established by X-ray crystal structures. Foremost, the molecular targets of DHQ chemical series were identified as the non-canonical poly(A) RNA polymerase-associated domain containing proteins 5 and 7 (PAPD5 and PAPD7). PAPD5 and PAPD7 are also known as terminal nucleotidyltransferase proteins 4B and 4A, respectively (TENT4B/A), orthologs of yeast Trf4 polypeptides.

The next year, a yeast three-hybrid (Y3H) screen also confirmed TENT4A and TENT4B as the direct targets of DHQ compounds [45]. The study showed that TENT4A and -B are essential for HBV RNA stabilization, and their catalytic domains are required for RG7834 interaction. A mutagenesis study showed that both the La-binding site and SLα, located in the PREα sub-element, are essential for DHQ-1-mediated HBV gene expression [33].

TENT4A/B post-transcriptionally incorporate nucleotides into the 3′ end of RNAs to regulate their maturation, stability, and activity [46,47,48,49,50,51,52,53]. They extend mRNA poly(A) tails with intermittent non-adenosine residues, usually guanosine, to generate what is commonly called “mixed tails” [50]. RNA tailing consists of the post-transcriptional addition of non-templated nucleotides to the 3′ end of various RNA types and is highly conserved. In mammalian cells, the addition of a poly(A) tail is known to either promote or delay RNA decay [54].

### 4.2. RG7834 Is a Potent HBV Inhibitor

The inhibition potency of RG7834 on HBV was evaluated, and a half-maximal effective concentration (EC_50_) of 1.6–8.7 nM was determined [33,43]. DHQ demonstrated selective sensitivity against HBV replication in comparison to 15 other viruses, including hepatitis C virus, herpes simplex virus-1, human cytomegalovirus, human rhinovirus, human immunodeficiency virus, dengue serotype 2 virus, West Nile virus, Influenza B, and the respiratory syncytial virus (all with an EC_50_ above 30,000) [33]. Hepatitis A virus (HAV) was shown recently to be inhibited by DHQ-1 as well (EC_50_ of 12.76), which is likely mediated via the TENT4A/B complex [55].

### 4.3. ZCCHC14 Bridges TENT4A/B Complex to Its Target PRE

It was unlikely that TENT4A and -B could directly interact with PRE for several reasons. First, they do not possess any obvious RNA binding domains. Second, their interaction with their target RNAs could be indirect, possibly via an intermediate protein that possesses an RNA-binding factor, with the possible leading candidate as the zinc finger CCHC-type containing protein 7 (ZCCHC7) [56]. This hypothesis was tested by the Y3H screen study mentioned above, but the knockdown of ZCCHC7 did not modulate HBV transcripts levels [45]. Therefore, other host intermediate proteins linking TENT4A/B to PRE were yet to be identified.

**Figure 2:**
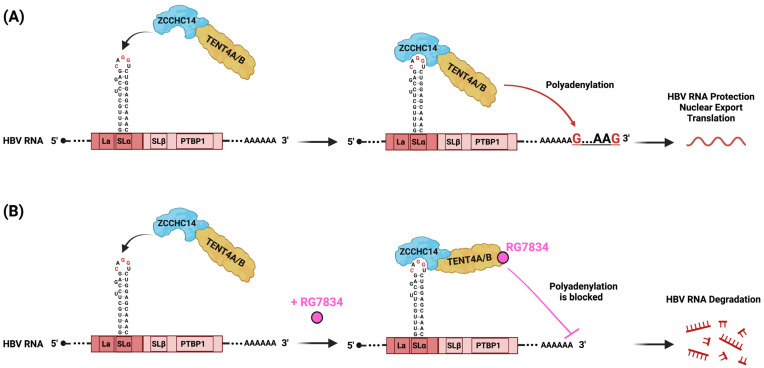
Schematic representation of PRE mode of action. (**A**) ZCCHC14 and TENT4A/B form a complex with the SLα pentaloop (CAGGU) of HPRE to initiate polyadenylation at the 3′ end, facilitating protection of HBV RNA against degradation, nuclear export to the cytoplasm, and translation. (**B**) RG7834 is a small molecule that specifically inhibits TENT4A/B enzymatic activity, thus blocking mixed tailing on the 3′ end of HBV RNA. This blockage leads to the degradation of HBV RNA by cellular nucleases, potently suppressing HBV replication both in vitro and in vivo. Figure created with Biorender.com.

Later, in a genome-wide CRISPR screen, the authors identified around 60 genes responsible for reducing HBsAg production. Among them was a large protein of 1086 amino acids called zinc finger CCHC-type containing protein 14 (ZCCHC14). This protein contains a sterile alpha motif (SAM) that could be an RNA-binding domain [57]. Interestingly, the study demonstrated that ZCCHC14 directly interacts with TENT4A/B and stabilizes HBsAg expression [58]. Later, another study testing different PRE constructs showed that ZCCHC14 binds precisely to the CNGGN-type pentaloop located in the stem–loop SLα of the PREα region [59] (Figure 2A). In the case of HBV genotype D, the pentaloop is composed of CAGGU nucleotides. TENT4 depletion resulted in the shortening of poly(A) tails from the PREα construct, but not from the PREß construct. It was previously shown that viral RNAs protected by the TENT4 mixed tail can counteract the CCR4-NOT (CNOT) deadenylase complex [50]. Interestingly, RG7834 can directly repress TENT4A/B polyadenylation, leading to the shortening of the poly(A) tail, followed by HBV mRNA destabilization and degradation in both the nucleus and the cytoplasm [60] (Figure 2B). When TENT4A/B was depleted, the half-lives of HBV pg, pc, preS1, and preS2/S mRNA were significantly reduced, except for HBx mRNA, which does not contain an SLα stem–loop.

In summary, HBV hijacks the ZCCHC14-TENT4 complex and recruits it to its PRE via a direct interaction of ZCCHC14 to the CAGGU pentaloop in SLα. This action stabilizes HBV RNA through RNA tailing and protects it from cellular decay factors, hence promoting HBsAg production. RG7834 represses TENT4A/B polyadenylation and leads to HBV RNA degradation [58,59,61].

## 5. PRE Is a Druggable Target for HBV Therapy

As discussed previously, RG7834, a DHQ-1 compound developed by Roche Pharma [43,44,45], induces the degradation of all HBV transcripts, except for the smallest 0.7 kb HBx mRNA [60]. This leads to the significant reduction of HBsAg, HBeAg, and HBV DNA in vitro and in vivo in HBV-infected uPA/SCID mice [44].

Therefore, RG7834 was further explored in preclinical and clinical studies. Its Phase 1 trial in healthy and chronic hepatitis B patients (NCT02604355) was halted due to its neurotoxicity. As an alternative, a series of RG7834 derivatives were designed, synthesized, and evaluated to create an improved small molecule version named *(2ʹS*, *6S)-1a*. It displayed in vitro anti-HBV activity comparable to RG7834 but with much reduced neurotoxicity [62]. Recently, a group of investigators chemically converted the RG7834 compound to a hepato-selective DHQ analog by adding an additional acid group into the RG7834 side chain. Their study revealed that the new compound is less neurotoxic than RG7834 in vivo, exhibiting a lower penetration of the blood–brain barrier due to its limited distribution in the bloodstream and, consequently, to other tissues [63].

Arbutus also developed a small molecule (AB452) targeting TENT4A/B, which showed efficacy in an animal model [33,63]. However, early clinical studies with this compound were halted to further evaluate its safety. Roche recently developed a Locked Nucleic Acid-based oligonucleotide program (RG LNA) targeting TENT4A and TENT4B and is currently under pre-clinical evaluation. A computational analysis study of HPRE revealed a novel and effective siRNA target site around nt 1317–1337 that is highly conserved [64]. The authors showed that a short hairpin RNA (shRNA) targeting this PRE site significantly decreased the expression of the reporter protein and specifically reduced cccDNA levels in transiently HBV-infected cells.

## 6. Considerations for PRE-Targeting Strategies in HBV Therapy

### 6.1. The Rationale of PRE-Targeting Strategies

The rationale of developing PRE-targeting strategies for HBV therapy relies on several considerations. First, PRE-targeting compounds, as described above, can effectively suppress HBV gene expression and thus replication. However, these compounds are difficult to formulate and have the potential for neurotoxicity. Second, this approach would likely require long-term therapy because it only suppresses viral gene expression and does not target the cccDNA. It is likely that stopping therapy would lead to viral rebound. Furthermore, it is unclear how PRE-targeting molecules would be more advantageous than other RNA-targeting strategies described below, with some already under clinical phase II evaluations.

### 6.2. Targeting HBV by RNA Interference Technology

Similar to the approach of targeting PRE, RNA interference technologies, like small interfering RNA (siRNAs), also specifically target cccDNA-derived HBV mRNA transcripts. After being delivered into hepatocytes, siRNAs hybridize with HBV mRNA, and the resulting double-stranded RNA is degraded [65].

The first anti-HBV siRNA developed was *ARC-520*, sponsored by Arrowhead Pharmaceuticals, which went through several clinical trials (NCT02452528, NCT02604212, NCT02604199, NCT02738008, NCT02065336, and NCT02577029) and showed high specificity, effectively reducing the production of HBV DNA and proteins [66]. A second-generation siRNA was developed with *ARC-521*, but trials (NCT02797522) were terminated because of potential safety issue with the delivery platform [67]. There are other anti-HBV siRNA from different companies that went through clinical trials: *ARB-1467* (TKM-HBV) in phase 2a (NCT02631096); *ARO-HBV* in phase 1/2a (NCT03365947); and *ALN-HBV*, *LUNAR^TM^ HBV*, *Hepbarna* (BB-HB-331), and *ARB-1740*. *VIR-2218*, an siRNA developed by VIR Biotechnology, has shown some clinical benefit [68].

Other nucleic acid-based technology, such as antisense oligonucleotides have been developed. *Bepirovirsen* (formerly known as IONIS-HBV_Rx_ and GSK3228836), developed by IONIS, demonstrated antiviral activities (NCT04449029/NCT02981602). Locked nucleic acid *RG6004* (RO7062931), developed by Roche, is currently under clinical trial (NCT03038113) [69].

It should be noted that, for most CHB patients, the use of RNA-targeting strategies alone has not led to a functional cure. Hence, more efforts are needed to develop strategies that address the stability of HBV RNAs in combination with additional methods targeting other stages of the HBV life cycle. The strategy of how PRE-targeting molecules would be used in combination with other antivirals, nucleos(t)ide analogues, capsid assembly modulators, or immunotherapies, remains to be clarified.

## 7. PRE-Like Elements in Other Viruses

PRE was also identified in the woodchuck hepatitis virus (WHV), a virus that belongs to the same *hepadnaviridae* family as HBV, and named WPRE [70]. WPRE consists of three sub-elements: WPREα, WPREß, and WPREγ. WPREα and WPREß are conserved between WHV and HBV, whereas WPREγ is distinctive and thought to be responsible for the greater activity of WPRE compared to HPRE. An investigation comparing bipartite HPRE and tripartite WPRE activities has shown that WPRE exhibits a two-to-three times greater activity than HPRE [70]. The structure of SLα was shown to be evolutionarily conserved in WHV, as well as the TENT4-mediated mechanism [33]. Other hepadnaviruses likely harbor similar genetic elements, suggesting an evolutionary adaptation of this family of viruses in their hosts.

RNA mixed poly(A) tailing, used for activation and viral replication, is not unique to HBV. It is also used by other viruses unrelated to the *hepadnaviridae* family. For instance, PRE was found within the *herpesviridae* family in the human cytomegalovirus (HCMV). Although the virus has a very different life cycle and tissue tropism than HBV, infecting epithelial, endothelial, fibroblast, and smooth muscle cells, it has been demonstrated that ZCCHC14 bridges TENT4 to HCMV RNA2.7 via the CNGGN pentaloop [58,59,60]. This interaction induces targeted mixed tailing, which protects the viral RNAs from cellular decay factors [59].

A functional viromic screen using a library of 3′ UTR viral segments made the connection that kobuvirus (a picornavirus) uses ZCCHC2, a different adapter of ZCCHC14, to recruit TENT4 in order to elongate poly(A) tails and prevent deadenylation [71], similarly to what HPRE does for HBV. TENT4-responsive RNA elements were also identified by authors in the norovirus and Saffold virus. This effect is similar to what HPRE does for HBV; however, the impact of these elements on the viral replication is still unknown.

Similar to PRE, the HIV-1 Rev response element (RRE) is a cis-acting RNA element that serves as a scaffold to coordinate the assembly of an RNP complex to mediate the nuclear export of un-spliced viral RNAs [25].

Genome-wide CRIPSR screens for HAV host factors found that, like for HBV, the ZCCHC14-TENT4 complex was also essential for viral replication and that it could be impaired by RG7834 treatment [55]. ZCCHC14 binds to stem–loop SL-Vb, within the 5′ UTR of HAV and recruits TENT4 [72]. However, RG7834 does not reduce the length of HAV poly(A) tail. Recently, a study identified a specific domain on ZCCHC14 that is crucial for binding to both HAV RNA and TENT4 [73].

## 8. PRE Application in Gene Therapy

Beyond antiviral therapy settings, PRE has an unexpected application in a different context. The WPRE has been incorporated into the design of gene delivery systems, as it significantly improves retroviral vector performance, ectopic gene expression, and lentiviral gene delivery [70,74]. Studies demonstrated that the incorporation of WPRE into different kinds of vectors (adeno-associated viruses, retroviruses, lentiviruses, and plasmid vectors) can enhance transgene expression and viral titers in various vectors and cell types [75,76]. An investigation of gene transfer strategies for neuronal and glial cells showed that optimization was obtained, with a minimum vector-associated toxicity, when using vectors containing PRE in plasmid and lentiviral vectors [77].

A study showed that the inclusion of WPRE enhanced AAV2-driven transduction of mouse and human retinas [78]. It was found that WPRE improves transgene expression and stability mediated by episomal vectors in CHO-K1 cells [79]. The inclusion of WPRE into baculovirus vectors provides a simple means to improve baculovirus-mediated gene expression in vertebrate cells [80].

This PRE-based technology has also been used in vaccine development. An in vitro and in vivo study on immunized mice showed that HPRE constructs enhanced antigen expression and DNA vaccine efficacy and immunogenicity [81]. Another study, which evaluated the protective efficacy of DNA vaccines against infectious bursal disease virus in chickens, discovered that WPRE enhanced its protective immune response [82].

## 9. Scope and Limitations of Current Experimental Systems to Study PRE

A variety of PRE constructs have been developed in the research field. The initial constructs used the production of HBsAg to assess the functionality of PRE [15]. Their use needs to be assessed in the presence of a control SV40-empty reporter. Later, various reporter constructs, such as luciferases, were generated [58,83]. These reporter constructs, defining PREα and PREß functional subregions of PRE, have been proven to be effective [59]. These constructs have been used successfully to investigate the functions and interactions of ZCCHC14 and TENT4A/B with PRE, as well as a screening platform to identify novel antivirals targeting the PRE. The existing DHQ and DHQ-E compounds [45] are useful as positive controls in assessing new PRE-targeting small molecules.

## 10. Conclusions and Future Directions

In this review, we discussed the characteristics and mode of action of PRE in stabilizing HBV mRNAs, resulting in the enhancement of viral gene expression. Other viruses also use a similar strategy to enhance their gene expression. The key feature contains cis-acting elements with a CNGGN pentaloop that is essential for the recruitment of the ZCCHC14-TENT4 complex. The mixed tailing by TENT4 protects viral RNAs and increases gene expression. HBV pg, pc, preS1, and preS2/2 RNAs are dependent on this complex for their maturation and stability. This complex plays a protective role for HBV mRNAs, and, in its absence, HBV RNAs are more prone to be degraded by the host RNA degrading and quality control machineries. Despite recent advances in deciphering the PRE mode of action and its interaction with host proteins, major gaps in our understanding of this complex function remain. Other cellular proteins that impact HBV mRNA levels are still yet to be identified. The discovery and characterization of PRE not only paves the way for potential HBV therapeutic development but also for other biomedical applications.

## Data Availability

Not applicable.
